# Not a Gorgon Sister

**Published:** 1918-08-10

**Authors:** 


### "Not a Gorgon Sister."

WE have received a set of verses, of which the following lines are samples, in praise of "My First Ward Sister." Soldier patients often overflow on the same theme, but the writer of the following is a nurse. If the last five lines are thought to show a falling-off, it is because they have suffered emendation:

A little wheel that doth never rest,
A little squirrel in a nest,
A loving heart that tireth never,
Thinking and planning for others ever.

A radiant smile that's never less,
No matter how hard her duties press;
Hands ever busy for others working,
Never the humblest duty shirking.

Her gentle words and sympathising touch
Were valued by her patients much;
Deftly and gently their wounds were dressed,
For care, as well as skill, her hands possessed.

A sister truly, she deserved her name:
Her discipline discipleship became.
Under her eye, like her her nurses grew
As true as steel, and thorough through and through.

